# Aripiprazole lauroxil 2-month formulation with 1-day initiation in patients hospitalized for an acute exacerbation of schizophrenia: exploratory efficacy and patient-reported outcomes in the randomized controlled ALPINE study

**DOI:** 10.1186/s12888-021-03420-x

**Published:** 2021-10-08

**Authors:** Henry A. Nasrallah, Peter J. Weiden, David P. Walling, Yangchun Du, Baiyun Yao, Sergey Yagoda, Amy Claxton

**Affiliations:** 1 University of Cincinnati College of Medicine, 260 Stetson Street, Suite 3200, Cincinnati, OH 45219 USA; 2 Alkermes, Inc., Waltham, MA USA; 3 CNS Network, LLC, Garden Grove, CA USA

**Keywords:** Antipsychotic drugs, Intramuscular injections, Quality of life, Dependency burden, Paliperidone palmitate

## Abstract

**Background:**

A randomized, controlled, phase 3b study (ALPINE) evaluated efficacy and safety of a 2-month formulation of aripiprazole lauroxil (AL) using a 1-day initiation regimen in patients hospitalized for an acute exacerbation of schizophrenia. Paliperidone palmitate (PP) was used as an active control. Exploratory endpoint assessments included severity of illness, positive and negative symptoms, quality of life, caregiver burden, and satisfaction with medication.

**Methods:**

Adults were randomly assigned to AL 1064 mg q8wk or PP 156 mg q4wk as inpatients, discharged after 2 weeks, and followed through week 25. Exploratory efficacy measures included the 3 original PANSS subscales, Clinical Global Impression−Severity (CGI-S) subscale, and caregiver Burden Assessment Scale. Exploratory patient-reported outcomes (PROs) included the Quality of Life Enjoyment and Satisfaction Questionnaire Short Form (Q-LES-Q-SF) and the Medication Satisfaction Questionnaire. Within-group changes from baseline through week 25 were analyzed for AL and PP separately. PROs were summarized based on observed data.

**Results:**

Of 200 patients randomized (AL, *n* = 99; PP, *n* = 101), 99 completed the study (AL, *n* = 56; PP, *n* = 43). For AL, PANSS subscale and CGI-S scores improved from baseline through week 25 (mean [SE] change from baseline at week 25: Positive, −7.5 [0.70]; Negative, −3.9 [0.46]; General, −11.8 [0.83]; CGI-S, −1.3 [0.12]). Caregiver burden also improved (mean [SD] changes from baseline at week 9: −8.4 [10.15]; week 25: −8.9 [12.36]). Most AL patients were somewhat/very satisfied with treatment at each timepoint (70.8%–74.7%); mean Q-LES-Q-SF total scores were stable in the outpatient period. For PP, results were similar: PANSS Positive, −7.3 (0.67); Negative, −3.6 (0.69); General, −10.9 (1.22); CGI-S, −1.4 (0.16); caregiver burden, week 9: −8.8 (11.89) and week 25: −9.2 (14.55); satisfaction with treatment, 64.7%–69.3%; and stable Q-LES-Q-SF scores.

**Conclusions:**

ALPINE patients initiating the 2-month AL formulation using the 1-day initiation regimen as inpatients and continuing outpatient care experienced schizophrenia symptom improvement, sustained patient satisfaction with medication, stable quality of life, and reduced caregiver burden. A similar benefit pattern was observed for PP. These results support the feasibility of starting either long-acting injectable in the hospital and transitioning to outpatient treatment.

**Trial registration:**

ClinicalTrials.gov identifier: NCT03345979 [trial registration date: 15/11/2017].

**Supplementary Information:**

The online version contains supplementary material available at 10.1186/s12888-021-03420-x.

## Background

Schizophrenia has remained a leading cause of disability worldwide over the past several decades [1], and data from the Global Burden of Disease Study indicate that both the incidence and disability-adjusted life years of schizophrenia have increased during this time [2]. Estimates of the annual US cost of schizophrenia range from 0.02% to 5.46% of the gross domestic product [3]. Most of that economic burden is indirect costs, including the cost of informal, unpaid care by caregivers [3].

Patients with schizophrenia experience functional disability across multiple domains associated with poor social and occupational outcomes [4, 5]. Although nearly half of patients treated using clinical practice guidelines may achieve symptomatic remission, far fewer will have a full functional recovery [6]. Because symptomatic remission may not predict functional recovery, a comprehensive evaluation of treatment efficacy in patients with schizophrenia requires assessment of patient-reported daily functioning and quality of life [5, 7, 8]. Lack of adherence to antipsychotic medication is significantly associated with poor outcomes in patients with schizophrenia [9–11], and patient satisfaction with medication has been identified as one of the determinants of medication adherence [12, 13]. Furthermore, low medication satisfaction is related to both lack of efficacy and drug side effects [13–16]. Therefore, assessment of patient satisfaction with medication in clinical trials is a valuable endpoint to measure given its relation to adherence, drug effectiveness, and patient outcomes.

Aripiprazole lauroxil (AL; ARISTADA®), a prodrug of aripiprazole, is a long-acting injectable (LAI) atypical antipsychotic indicated for the treatment of schizophrenia in adults [17, 18]. The randomized, controlled, phase 3b Aripiprazole Lauroxil and Paliperidone palmitate: INitiation Effectiveness (ALPINE) study evaluated the efficacy and safety of a 2-month formulation of AL started with a 1-day initiation regimen during hospitalization for an acute exacerbation of schizophrenia [19]. At the time the study began, the 2-month AL formulation had recently become available and the 1-day alternative to 3-week oral aripiprazole initiation was under US Food and Drug Administration review. The goal of the ALPINE study was to provide clinical data on the efficacy and safety of these AL formulations in a treatment setting consistent with the use of LAIs in clinical practice. Because AL was a relatively new LAI option, paliperidone palmitate (PP; INVEGA SUSTENNA®), an effective, widely used LAI that is familiar to clinicians [20–22], was chosen as an active control. The primary endpoints were previously reported [19]: Positive and Negative Syndrome Scale (PANSS) [23] total scores improved from baseline at primary (week 4) and secondary (weeks 9 and 25) endpoints for patients treated with AL or PP in the ALPINE study. However, outcomes exploring broader domains relevant to patients’ full functional recovery have not been reported. Therefore, exploratory efficacy endpoints and caregiver- and patient-reported outcomes (PROs) from the ALPINE study are reported here.

## Methods

The ALPINE study (ClinicalTrials.gov identifier: NCT03345979 [trial registration date: 15/11/2017]) was designed and carried out in accordance with the principles of Good Clinical Practice that have their origin in the Declaration of Helsinki and its amendments [24] and in accordance with local regulations and International Council for Harmonisation guidelines [25]. The study protocol and amendment were approved by the independent ethics committee/institutional review board for each study site. All participants provided written informed consent before any study-specific procedures were conducted. The first patient visit occurred 15 November 2017, and the last visit occurred 12 March 2019.

### Patients

Patients were adults (age 18–65 years) with a primary diagnosis of schizophrenia and an acute exacerbation or relapse of symptoms requiring hospitalization. Enrollment criteria have been published previously [19]. Key criteria included a PANSS total score of 80–120, with scores of ≥4 for ≥2 of the following Positive subscale items: 1 (delusions), 2 (conceptual disorganization), 3 (hallucinatory behavior), or 6 (suspiciousness/persecution), and a Clinical Global Impression-Severity (CGI-S) [26] score ≥4. Patients were excluded if they had a primary diagnosis other than schizophrenia, current risk of suicide, history of treatment resistance, or recent LAI treatment.

### Study design

**Fig. 1:**
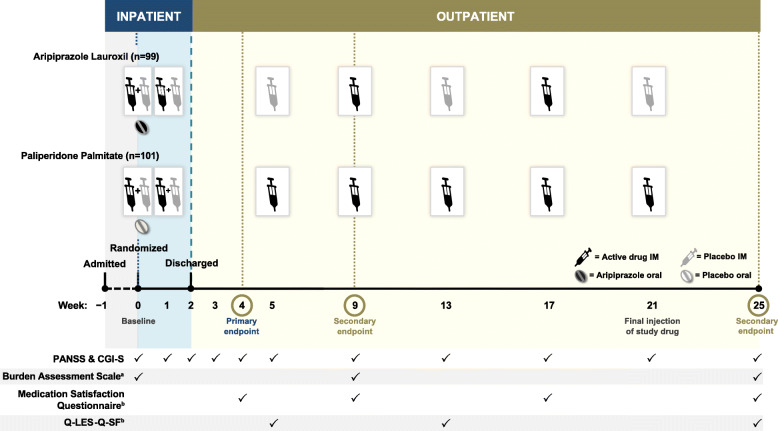
ALPINE Study Design and Exploratory Outcome Schedule. ^a^Caregiver-reported outcome; the baseline assessment for the Burden Assessment Scale was completed during the screening period. ^b^Patient-reported outcome. CGI-S, Clinical Global Impression-Severity; IM, intramuscular; PANSS, Positive and Negative Syndrome Scale; Q-LES-Q-SF, Quality of Life Enjoyment and Satisfaction Questionnaire–Short Form

After consent, prior antipsychotic medications were discontinued in the hospital during a 2–5 day washout period. Patients were then randomly assigned (1:1) to start 1 of the 2 LAIs (AL or PP) on study day 1, with blinding maintained from randomization until the end of study treatment (Fig. 1) [19]. Patients without prior exposure to AL, PP, or both received a test dose of the corresponding oral antipsychotic (aripiprazole or risperidone) to establish tolerability. Randomization was stratified by prior history of exposure to aripiprazole and/or risperidone/paliperidone [19]. The inpatient stay included the screening period and at least the first 2 weeks of double-blind treatment. After discharge, participants were followed as outpatients for the remainder of the 25-week treatment period. Outpatient visits were scheduled at study post-randomization weeks 3, 4, and 5 and then every 4 weeks through week 25.

AL was initiated on study day 1 using a 1-day regimen that included a single gluteal injection of AL NanoCrystal® Dispersion (AL_NCD_; ARISTADA INITIO®) plus a single 30 mg oral aripiprazole tablet. The AL_NCD_ formulation has a faster dissolution time and results in a more rapid rise in plasma aripiprazole concentrations compared with AL [17, 27]. However, because AL_NCD_ is also an LAI, a single, one-time 30 mg dose of oral aripiprazole is administered on the same day as the AL_NCD_ injection to eliminate any remaining delay in achieving aripiprazole plasma concentrations that are associated with a therapeutic effect [17]. AL 1064 mg was administered in a gluteal injection on day 8 and every 8 weeks thereafter (for a total of 3 injections of AL 1064 mg); no oral aripiprazole was administered with AL 1064 injections. PP was started with a PP initiation dose of 234 mg on day 1, followed by PP 156 mg on day 8 (both deltoid injections). PP 156 mg was administered in gluteal injections every 4 weeks thereafter (for a total of 6 injections of PP 156 mg). Because pharmacokinetic analyses indicate that steady-state plasma aripiprazole concentrations after administration of AL 1064 mg every-2-months are lower than those associated with the highest AL dose (882 mg monthly) [28], the intermediate approved PP dose of 156 mg monthly [29] was selected as the comparator. After the day 1 randomization visit, no additional oral antipsychotic was allowed for the rest of the study, including as rescue medication.

Patients and their caregivers, investigators, and all study site personnel (except the trained pharmacists who administered the study drug but made no assessments) were blinded to treatment assignment throughout the study; placebos were used to maintain blinding. The PP group received an oral placebo tablet on day 1 to match the oral dose of aripiprazole in the AL initiation regimen (Fig. 1). Because AL was initiated using a gluteal injection (AL_NCD_) and PP was initiated using a deltoid injection (PP 234 mg) per protocol, placebo injections were used at the opposite injection site during initiation. The AL group also received placebo injections at weeks 5, 13, and 21 to match the PP dosing schedule. To prevent functional unblinding, postbaseline prolactin levels were blinded to investigators and study site staff until the end of the study.

### Assessments

Exploratory efficacy endpoints included PANSS Positive, Negative, and General Psychopathology subscale scores and CGI-S scale scores. PANSS and CGI-S were administered as inpatient assessments at screening, randomization (week 0), day 4, and weeks 1 and 2 and then at each outpatient visit (Fig. 1). The possible scoring ranges for the PANSS subscales are 7–49 for the 7-item Positive and 7-item Negative subscales and 16–112 for the 16-item General Psychopathology subscale [23]. Higher scores indicate greater burden of symptoms for each subscale. The CGI-S scale assesses severity of illness in a single item scored from 1 (normal, not at all ill) to 7 (among the most extremely ill patients) [26]. The CGI-S was the final assessment administered during the session, and investigators were instructed to rate global severity of illness based on clinical judgment and experience.

Caregiver burden was assessed using the Burden Assessment Scale [30], a 19-item instrument assessing consequences of caring for individuals with severe mental disorders. The Burden Assessment Scale was completed by a family member or friend who cared for the patient but was not a professional caregiver. Each Burden Assessment Scale item evaluates a possible area of impact (eg, “had financial problems” or “missed days at work [or school]”), with each item scored on a 4-point Likert scale as follows: 1 (not at all), 2 (a little), 3 (some), or 4 (a lot). The possible total score ranges from 19 (lowest burden) to 76 (highest burden); a total score of approximately 38 would indicate “a little” burden overall (averaged across items), and a score of 57 would indicate “some” burden.

Patient-reported outcomes were administered only during the outpatient period because of concern about the reliability of self-report assessments by patients with acute illness [31, 32]. The PROs included a modified version of the Medication Satisfaction Questionnaire (MSQ) [33] and the Quality of Life Enjoyment and Satisfaction Questionnaire–Short Form (Q-LES-Q-SF) [34]. The modified MSQ is a 3-item measure of satisfaction with injectable medication administered to patients with schizophrenia. The 3 items include satisfaction with current injectable medication, preference for current injectable medication or prior oral medication, and level of side effects of current injectable versus previous oral medication. Each item has 5 possible response levels (for example, “How satisfied are you with your current injectable medication?”; possible responses: very dissatisfied [1], somewhat dissatisfied [2], neither [3], somewhat satisfied [4], or very satisfied [5]).

The Q-LES-Q-SF is a 16-item scale that assesses overall enjoyment and satisfaction with physical health, mood, work, household and leisure activities, social and family relationships, daily functioning, sexual life, economic status, overall well-being, and medications [34]. Each item evaluates satisfaction in an area of daily functioning over the past week, scored from 1 (very poor) to 5 (very good). The Q-LES-Q-SF total score is calculated by summing the first 14 items and has a possible scoring range of 14 to 70. Items 15 (Satisfaction with Medication) and 16 (Overall Life Satisfaction) are stand-alone items reported separately. The timing of PRO and caregiver burden assessments is shown in Fig. 1.

### Statistical analysis

All patients enrolled in ALPINE who received ≥1 dose of study drug and had ≥1 postbaseline PANSS assessment were included in the current exploratory efficacy analysis. Patients who provided data for a given endpoint were included in the analysis of that endpoint; all analyses were based on observed data without imputation of missing data. The ALPINE study was not designed for a statistical comparison between treatment groups for exploratory efficacy endpoints. Therefore, exploratory efficacy endpoints were assessed separately for AL and PP without between-group comparisons, and results are presented by treatment group. No conclusions about relative efficacy for AL versus PP can be drawn. All endpoints were characterized descriptively using SAS® (V9.4) statistical software (SAS Institute Inc., Cary, NC). No formal statistical testing or sample size calculations were performed.

Mean (SE) score and mean (SE) change in score from baseline (week 0) on the PANSS Positive, Negative, and General Psychopathology subscales and CGI-S scale through week 25 were summarized by visit for AL or PP treatment groups separately. Median scores on the CGI-S scale were also assessed.

Burden Assessment Scale total scores were summarized using descriptive statistics for the AL and PP treatment groups separately. Mean (SD) total score and change from baseline (screening visit) at each timepoint are presented. A subgroup analysis was also conducted in patients who completed the study and whose caregiver provided Burden Assessment Scale data at both baseline and week 25.

Responses to each modified MSQ item were grouped in 3 categories (very or somewhat dissatisfied; somewhat or very satisfied; neither). For all patients, number and percentage of patients in each response category were presented by timepoint for AL and PP treatment groups. In a separate analysis of patients with MSQ responses at all 4 timepoints, the stability of individual patients’ level of satisfaction with current injectable medication over time was assessed. The numbers and percentages of patients who responded within the same category at each of the 4 timepoints (defined as “stable” for this analysis) versus those whose responses shifted from 1 category of response to another were calculated. Mean (SD) score for level of satisfaction with current injectable medication was then calculated at each timepoint separately for patients with stable responses and for patients whose responses shifted.

Mean (SD) Q-LES-Q-SF total score and Satisfaction with Medication and Overall Life Satisfaction item scores were calculated for AL and PP treatment groups at weeks 5, 13, and 25. Q-LES-Q-SF total score and Satisfaction with Medication and Overall Life Satisfaction item scores were also summarized for all patients who completed the study and had Q-LES-Q-SF assessments at weeks 5 and 25.

## Results

### Patients

Patient disposition and demographic and baseline characteristics were previously reported in the primary ALPINE publication [19]. Briefly, 200 patients were randomized to treatment in ALPINE (AL, *n* = 99; PP, *n* = 101; Supplemental Fig. 1). All 99 patients assigned to AL received ≥1 dose of AL; 96 patients had ≥1 postbaseline PANSS assessment and were included in the efficacy analysis. The 25-week completion rate was 56.6% for the AL group. All 101 patients randomized to PP received ≥1 dose of PP, and 99 had ≥1 postbaseline PANSS assessment and were included in the efficacy analysis. The study completion rate for the PP group was 42.6%.

As previously reported [19], approximately 75% of patients in each treatment group were male (AL, *n* = 73, 73.7%; PP, *n* = 76, 75.2%) and the majority of patients were Black or African American (AL, *n* = 72, 72.7%; PP, *n* = 78, 77.2%). Mean (SD) age at baseline was 43.5 (9.67) years for the AL group and 43.4 (10.83) years for the PP group. Mean (SD) baseline PANSS total score was 94.1 (9.04) for the AL group and 94.6 (8.41) for the PP group. Most caregivers identified themselves as friends (AL: *n* = 28, 31%; PP: *n* = 29, 31%), parents (AL: *n* = 18, 20%; PP: *n* = 14, 15%), or spouses/partners (AL: n = 14, 16%; PP: *n* = 10, 11%) of the patient; the remainder included siblings, children, aunts/uncles/cousins, or residential setting representatives. Caregivers most commonly reported having known the patient for the length of the patient’s lifetime (AL: *n* = 35, 39%; PP: *n* = 36, 38%), for 5 or more years (AL: *n* = 25, 28%; PP: *n* = 21, 22%), or for 1 to 5 years (AL: n = 18, 20%; PP: *n* = 32, 34%).

Additional results are presented below first for patients receiving the treatment of interest (AL) and then for those receiving the active comparator (PP) to illustrate changes in the outcome variables with 2 treatments with known efficacy.

### Aripiprazole lauroxil treatment outcomes

#### Exploratory efficacy outcomes

**Fig. 2:**
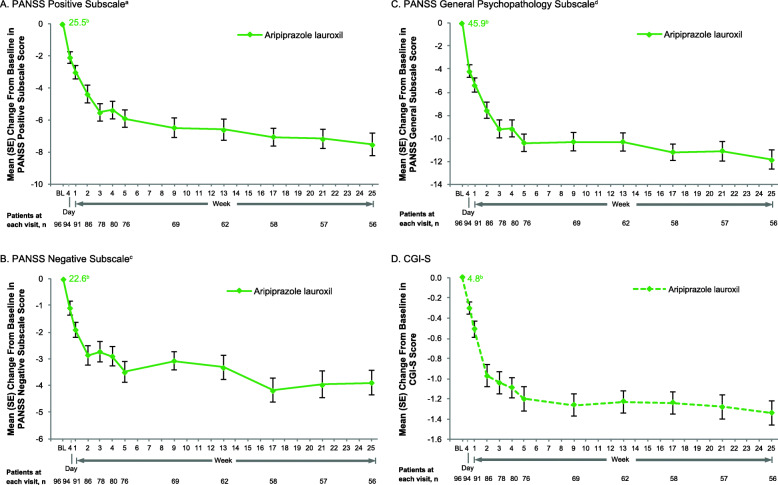
Change From Baseline in PANSS Subscale and CGI-S Scores With Aripiprazole Lauroxil, Observed Cases. **A.** PANSS Positive Subscale^a^. **B.** PANSS Negative Subscale^c^. **C.** PANSS General Psychopathology Subscale^d^. **D.** CGI-S^a^. PANSS Positive subscale score calculated as sum of items P1–7 (Delusions, Conceptual disorganization, Hallucinatory behavior, Excitement, Grandiosity, Suspiciousness, Hostility); scoring range, 7–49^b^. Mean baseline score is indicated at the first timepoint; mean (SE) change from baseline scores are indicated at each subsequent timepoint^c^. PANSS Negative subscale score calculated as sum of items N1–7 (Blunted affect, Emotional withdrawal, Poor rapport, Passive-apathetic social withdrawal, Difficulty in abstract thinking, Lack of spontaneity & flow of conversation, Stereotyped thinking); scoring range, 7–49^d^. PANSS General Psychopathology subscale score calculated as sum of items G1–16 (Somatic concern, Anxiety, Guilt feelings, Tension, Mannerisms & posturing, Depression, Motor retardation, Uncooperativeness, Unusual thought content, Disorientation, Poor attention, Lack of judgment & insight, Disturbance of volition, Poor impulse control, Preoccupation, Active social avoidance); scoring range, 16–112. BL, baseline; CGI-S, Clinical Global Impression−Severity; PANSS, Positive and Negative Syndrome Scale

PANSS Positive, Negative, and General Psychopathology subscale scores improved from baseline throughout the study for the AL treatment group (Fig. 2A–C). Change from baseline in each subscale score was numerically greatest over the first ~3 weeks of treatment, and improvement was observed through week 25 (no statistical tests among timepoints were performed). The mean (SE) change from baseline at week 25 was −7.5 (0.70) in the Positive subscale score (baseline mean [SD]: 25.5 [3.80]), −3.9 (0.46) in the Negative subscale score (baseline: 22.6 [3.74]), and −11.8 (0.83) in the General Psychopathology subscale score (baseline: 45.9 [5.50]). CGI-S scores also improved from a baseline mean (SD) of 4.8 (0.65) to 3.4 (0.74) at week 25 of AL treatment (mean [SE] change from baseline, −1.3 [0.12]; Fig. 2D). The median CGI-S score at baseline was 5 (markedly ill; *n* = 96) for the AL group; at week 25, the median CGI-S score was 3 (mildly ill; *n* = 56).

### *Caregiver- and patient-reported outcomes*

Mean (SD) Burden Assessment Scale total score at baseline was 40.8 (14.17) for the AL group (Table 1). Mean (SD) improvement in total score observed at week 9 (change from baseline, −8.4 [10.2]) was maintained at week 25 (−8.9 [12.4]). Responses from the caregivers of AL patients who completed the study and had both baseline and week 25 assessments were similar to those from the caregivers of all AL patients (Table 1).

**Table 1:** Burden Assessment Scale^a^, Aripiprazole Lauroxil Group

	Baseline	Week 9	Week 25
**All patients (observed cases)**			
Total score, patients, n	88	53^b^	43^c^
Mean (SD)	40.8 (14.2)	32.7 (13.1)	31.8 (11.9)
Change from baseline, mean (SD)	–	−8.4 (10.2)	−8.9 (12.4)
**Patients who completed 25 weeks of treatment**^**d**^			
Total score, patients, n	42	40	42
Mean (SD)	40.6 (15.1)	33.1 (13.8)	31.7 (12.0)
Change from baseline, mean (SD)	–	−8.0 (9.8)	−8.9 (12.4)

^a^Range, 19–76

^b^Data from 52 patients contributed to the change from baseline value at week 9

^c^Data from 42 patients contributed to the change from baseline value at week 25

^d^All patients who had Burden Assessment Scale data at baseline and week 25

**Fig. 3:**
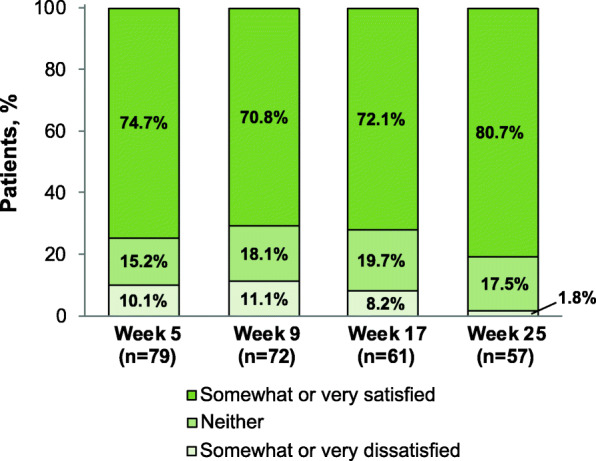
Medication Satisfaction Questionnaire Satisfaction with Current Injectable Medication Item^a^, Aripiprazole Lauroxil Group^a^. Patients were asked, “How satisfied are you with your current injectable medication?” Possible responses were very dissatisfied, somewhat dissatisfied, neither, somewhat satisfied, or very satisfied

**Fig. 4:**
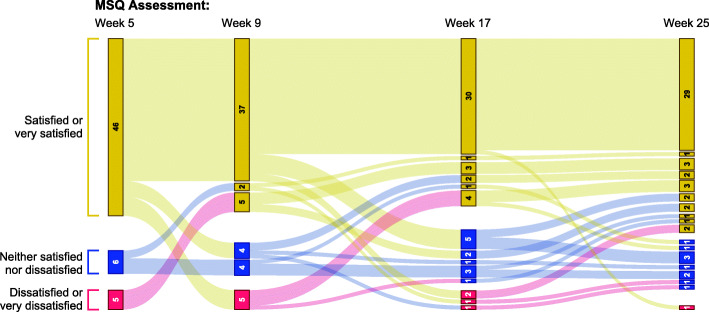
Shifts in Satisfaction with Current Injectable Medication, Aripiprazole Lauroxil Group. Gold bars represent patients who were satisfied or very satisfied with their current injectable medication at a given assessment; blue bars indicate patients who were neither satisfied nor dissatisfied, and red bars indicate patients who were dissatisfied or very dissatisfied. Path color indicates patients’ level of satisfaction at the previous MSQ assessment. The width of each bar and path is proportional to the number of patients represented. For example, 5 patients who were satisfied or very satisfied with treatment at week 5 reported that they were dissatisfied or very dissatisfied with treatment at week 9; of those 5, 4 were again satisfied or very satisfied at week 17 while one was neither satisfied nor dissatisfied. All 5 patients who were dissatisfied or very dissatisfied with treatment at week 5 were satisfied or very satisfied at week 9. AL patients who completed MSQ at all 4 timepoints are included; *n* = 57

The majority of AL-treated patients who provided responses on the modified MSQ at ≥1 timepoint were somewhat satisfied or very satisfied with their current injectable medication (70.8%–80.7% across timepoints; Fig. 3). Mean (SD) satisfaction (scored from 1 [very dissatisfied] to 5 [very satisfied]) increased from 3.95 (1.12) at week 5 to 4.25 (0.81) at week 25. Responses to MSQ items 2 (preference for current or previous medication) and 3 (level of side effects with current versus previous medication) are presented for the AL group in Supplemental Fig. 2. For AL patients with MSQ data at all 4 assessments (*n* = 57), changes in patients’ levels of satisfaction with current injectable medication over the 4 timepoints are illustrated in Fig. 4.

Mean total Q-LES-Q-SF scores were consistent across assessments at weeks 5, 13, and 25 both overall and for patients who completed the assessment at all 3 timepoints (Table 2). Patients’ mean Q-LES-Q-SF Satisfaction with Medication and Overall Life Satisfaction scores remained between good (4) and fair (3) throughout the study.

**Table 2:** Quality of Life Enjoyment and Satisfaction Questionnaire–Short Form Scores^a^ Over Time, Aripiprazole Lauroxil Group

	Week 5	Week 13	Week 25
**All patients (observed cases)**			
Total score, n	73	65	57
Mean (SD)	48.7 (11.2)	48.7 (10.1)	49.0 (11.5)
Satisfaction with Medication, n	67	63	52
Mean (SD)	3.9 (1.0)	3.8 (1.0)	3.8 (0.9)
Overall Life Satisfaction, n	73	65	57
Mean (SD)	3.9 (1.0)	3.7 (0.9)	3.8 (1.0)
**Patients who completed 25 weeks of treatment**^b^			
Total score, n	57	57	57
Mean (SD)	49.2 (10.3)	49.1 (9.3)	49.0 (11.5)
Satisfaction with Medication, n	52	55	52
Mean (SD)	4.0 (0.8)	3.9 (0.9)	3.8 (0.9)
Overall Life Satisfaction, n	57	57	57
Mean (SD)	3.8 (0.9)	3.7 (0.9)	3.8 (1.0)

^a^Maximum total score: 70

^b^All patients who had Quality of Life Enjoyment and Satisfaction Questionnaire–Short Form data at weeks 5 and 25

### Paliperidone palmitate treatment outcomes

#### Exploratory efficacy outcomes

**Fig. 5:**
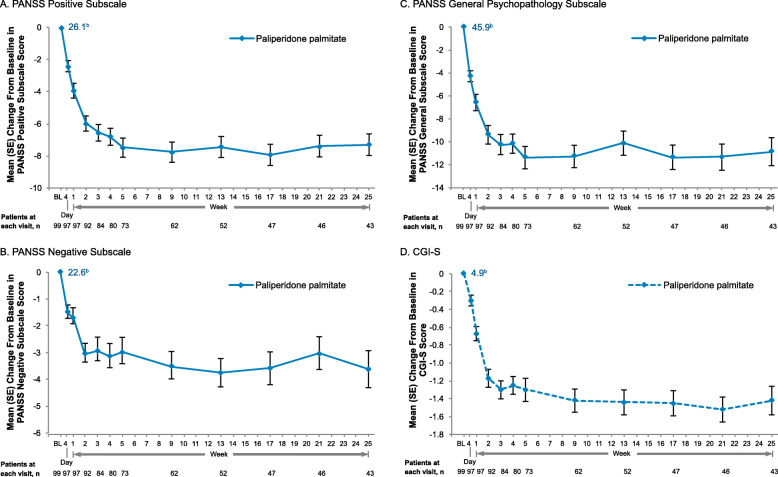
Change From Baseline in PANSS Subscale and CGI-S Scores With Paliperidone Palmitate, Observed Cases. **A.** PANSS Positive Subscale^a^.  **B.** PANSS Negative Subscale^c^. **C.** PANSS General Psychopathology Subscale^d^. **D.** CGI-S^a^. PANSS Positive subscale score calculated as sum of items P1–7 (Delusions, Conceptual disorganization, Hallucinatory behavior, Excitement, Grandiosity, Suspiciousness, Hostility); scoring range, 7–49^b^. Mean baseline score is indicated at the first timepoint; mean (SE) change from baseline scores are indicated at each subsequent timepoint^c^. PANSS Negative subscale score calculated as sum of items N1–7 (Blunted affect, Emotional withdrawal, Poor rapport, Passive-apathetic social withdrawal, Difficulty in abstract thinking, Lack of spontaneity & flow of conversation, Stereotyped thinking); scoring range, 7–49^d^. PANSS General Psychopathology subscale score calculated as sum of items G1–16 (Somatic concern, Anxiety, Guilt feelings, Tension, Mannerisms & posturing, Depression, Motor retardation, Uncooperativeness, Unusual thought content, Disorientation, Poor attention, Lack of judgment & insight. Disturbance of volition, Poor impulse control, Preoccupation, Active social avoidance); scoring range, 16–112. BL, baseline; CGI-S, Clinical Global Impression−Severity; PANSS, Positive and Negative Syndrome Scale.

Improvement in PANSS Positive, Negative, and General Psychopathology subscale scores was observed over the first several weeks of treatment with the active control PP and through the study period (Fig. 5A–C). The mean (SE) change from baseline at week 25 was − 7.3 (0.67) in the Positive subscale score (baseline mean [SD]: 26.1 [3.36]), −3.6 (0.69) in the Negative subscale score (baseline: 22.6 [3.63]), and −10.9 (1.22) in the General Psychopathology subscale score (baseline: 45.9 [5.32]). Mean (SD) CGI-S score improved from a baseline of 4.9 (0.65 [markedly ill]) to 3.6 (1.01 [mildly to moderately ill]) at week 25 of PP treatment (mean [SE] change from baseline, −1.4 [0.16]; Fig. 5D). The median CGI-S score at baseline was 5 (markedly ill; *n* = 99) for the PP group; at week 25, the median CGI-S score was 3 (mildly ill; *n* = 43).

#### Caregiver- and patient-reported outcomes

Mean (SD) baseline Burden Assessment Scale total score was 38.9 (14.40) (Table 3). Mean (SD) Burden Assessment Scale total score decreased with PP treatment, with most of that change observed between baseline and week 9 (change from baseline, − 8.8 [11.9]). Burden Assessment Scale scores for PP patients who completed the study (and provided responses at baseline and week 25) were similar to those for all PP patients (Table 3).

**Table 3:** Burden Assessment Scale,^a^ Paliperidone Palmitate Group

	Baseline	Week 9	Week 25
**All patients (observed cases)**			
Total score, patients, n	96	46	33
Mean (SD)	38.9 (14.4)	31.3 (13.0)	31.5 (13.0)
Change from baseline, mean (SD)	–	−8.8 (11.9)	−9.2 (14.6)
**Patients who completed 25 weeks of treatment**^**b**^			
Total score, patients, n	33	31	33
Mean (SD)	40.6 (13.5)	30.6 (13.2)	31.5 (13.0)
Change from baseline, mean (SD)	–	−9.9 (13.2)	−9.2 (14.6)

^a^Range: 19–76

^b^All patients who had Burden Assessment Scale data at baseline and week 25

**Fig. 6:**
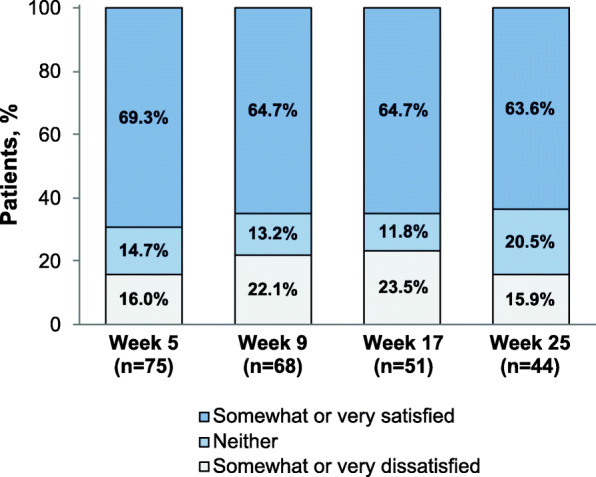
Medication Satisfaction Questionnaire^a^ Satisfaction with Current Injectable Medication Item, Paliperidone Palmitate Group. ^a^Patients were asked, “How satisfied are you with your current injectable medication?” Possible responses were very dissatisfied, somewhat dissatisfied, neither, somewhat satisfied, or very satisfied

**Fig. 7:**
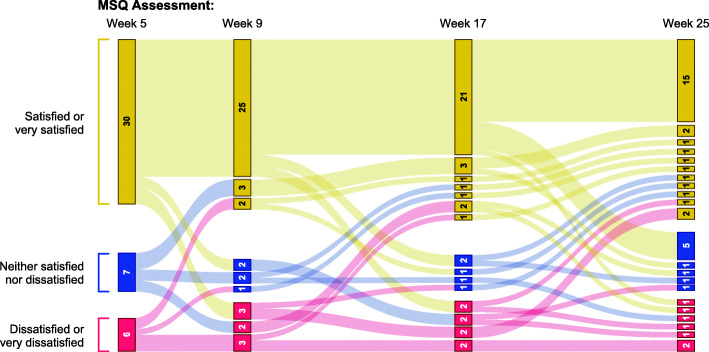
Change in Satisfaction with Current Injectable Medication, Paliperidone Palmitate Group. Gold bars represent patients who were satisfied or very satisfied with their current injectable medication at a given assessment; blue bars indicate patients who were neither satisfied nor dissatisfied, and red bars indicate patients who were dissatisfied or very dissatisfied. Path color indicates patients’ level of satisfaction at the previous MSQ assessment. The width of each bar and path is proportional to the number of patients represented. PP patients who completed MSQ at all 4 timepoints are included; *n* = 43

The majority of patients in the PP group who had modified MSQ data at ≥1 timepoint were somewhat satisfied or very satisfied with their current injectable medication at each timepoint (63.6%–69.3%; Fig. 6). Mean (SD) satisfaction scores ranged from 3.72 (1.34) at week 9 to 3.91 (1.31) at week 25 (where a score of 4 indicated somewhat satisfied). Responses to MSQ items 2 and 3 are presented for the PP group in Supplemental Fig. 3. For those PP patients with MSQ scores at each timepoint (*n* = 43), shifts in levels of satisfaction with current injectable medication over the 4 MSQ assessment periods are displayed in Fig. 7.

Mean total Q-LES-Q-SF scores were stable across assessments (Table 4), and mean Satisfaction with Medication and Overall Life Satisfaction scores remained between good (4) and fair (3) throughout the study. Similar results were observed for patients who provided Q-LES-Q-SF data at each assessment period (Table 4).

**Table 4:** Quality of Life Enjoyment and Satisfaction Questionnaire–Short Form Scores^a^ Over Time, Paliperidone Palmitate Group

	Week 5	Week 13	Week 25
**All patients (observed cases)**			
Total score, n	67	59	45
Mean (SD)	49.6 (10.1)	48.0 (12.8)	49.6 (12.3)
Satisfaction with Medication, n	55	52	44
Mean (SD)	3.6 (0.9)	3.7 (1.1)	3.6 (1.2)
Overall Life Satisfaction, n	67	59	45
Mean (SD)	3.9 (1.0)	3.5 (1.2)	3.6 (1.2)
**Patients who completed 25 weeks of treatment**^**b**^			
Total score, n	45	45	45
Mean (SD)^a^	49.2 (10.3)	47.8 (13.5)	49.6 (12.3)
Satisfaction with Medication, n	36	41	44
Mean (SD)	3.6 (0.9)	3.7 (1.2)	3.6 (1.1)
Overall Life Satisfaction, n	45	45	45
Mean (SD)	3.8 (1.0)	3.5 (1.3)	3.6 (1.2)

^**a**^Maximum total score: 70

^b^All patients who had Quality of Life Enjoyment and Satisfaction Questionnaire–Short Form data at weeks 5 and 25

## Discussion

Efficacy for treating symptoms of schizophrenia based on significant improvement from baseline in PANSS total score at weeks 4, 9, and 25 was observed in both the AL and PP treatment groups in the ALPINE study [19]. Results of some previously published analyses have suggested that the Positive and Negative subscales may be more sensitive to change over time or that antipsychotic medications may work differentially on positive versus negative symptoms [35–38]. Therefore, we examined the PANSS subscales in the current analysis of ALPINE study data. In this analysis of ALPINE exploratory efficacy endpoints, PANSS Positive, Negative, and General Psychopathology subscale results support ALPINE primary and secondary efficacy findings and are consistent with PANSS subscale results for AL 441 mg q4wk and 882 mg q4wk dose regimens in the AL pivotal study [39, 40]. In ALPINE, the AL 1064 mg q8wk regimen was associated with a mean (SE) change from baseline of −3.9 (0.46) on the Negative subscale score and change from baseline on the Positive subscale score was −7.5 (0.70). For each PANSS subscale, and similarly for the CGI-S, the greatest improvement with AL was observed in the first 2–3 weeks of treatment, followed by more gradual improvement during the ALPINE outpatient period (Fig. 2). A similar pattern of improvement in these exploratory efficacy measures was observed in the PP active control group (Fig. 5).

Treatment goals for patients with schizophrenia include maximizing adaptive functioning and quality of life, in addition to symptom reduction [4]. In this analysis of the exploratory Q-LES-Q-SF [41] endpoint, enjoyment and satisfaction in various areas of daily life were stable through the outpatient period, with mean scores ranging from 48.7 to 49.2 out of 70 over 25 weeks of AL treatment (Table 2). Patient-reported functioning results for the subgroup of patients who completed the study were similar to results for the full Q-LES-Q-SF analysis population. A community norm for Q-LES-Q-SF total score has been calculated as 58.1 for a group of volunteer subjects (*N* = 67) without mental or medical illness (mean age, 32.4 years; 65.8% women, and majority Caucasian) [42], but normative scores for patients with schizophrenia have not been reported. It should also be noted that the reliability of PROs may be limited in patients with schizophrenia, particularly in those with cognitive disturbance or lack of insight [31, 32, 43]. Although severely impaired patients may be unreliable respondents, some studies suggest that self-reported measures can be reliable in patients with less severe cognitive or insight impairment [44, 45]. In previous studies [20, 46–48], AL and PP treatment have both been associated with improved functional outcomes, including the PANSS Prosocial subscales (AL), the Personal and Social Performance scale (AL and PP), and the Social and Occupational Functioning Assessment Scale (PP).

Positive outcomes in patients with schizophrenia may be critically dependent on attitudes toward medication [49]. Patients report that a lack of perceived benefit and issues with the tolerability of their antipsychotic regimen are among reasons for low satisfaction and nonadherence to medications for schizophrenia [50–52], and poor treatment adherence is associated with an increased risk of relapse and rehospitalization [10, 53–55]. In ALPINE, a large majority of patients were somewhat or very satisfied with their LAI medication at each point in the 25-week treatment period. Rates of treatment satisfaction were as high as 80.7% for AL-treated patients (Fig. 3), and approximately half of AL patients who responded at all 4 timepoints were satisfied or very satisfied at all assessment timepoints (Fig. 4). The majority of patients treated with PP also were somewhat or very satisfied with their medication at each assessment (Fig. 6). Notably, the use of placebo injections to maintain blinding in the ALPINE study substantially increased the number of injections administered, particularly for patients receiving AL, and therefore the current analysis may underestimate satisfaction that might be associated with the AL q8wk regimen.

There were several additional limitations in the analysis of exploratory efficacy and caregiver- and patient-reported outcomes from the ALPINE study. First, the study was not powered for a direct comparison between the AL and PP treatment groups. The blinded PP arm provided an active control with known efficacy to inform the assessment of the efficacy and safety of the 2-month AL formulation and 1-day initiation regimen while avoiding the use of placebo treatment in patients requiring pharmacotherapy for an acute exacerbation of schizophrenia. Interpretation of these findings is also limited in the absence of a placebo arm. Finally, because the data presented here were collected in patients who met the specific ALPINE study inclusion and exclusion criteria, the results from this phase 3b clinical trial may not be generalizable to the broader population of hospitalized patients with schizophrenia who are initiating LAIs.

## Conclusions

In the ALPINE study, patients who initiated AL or PP in the hospital and continued treatment during outpatient care experienced improvement in positive, negative, and global schizophrenia symptoms, demonstrating the effectiveness of the AL 2-month dosing regimen started with a 1-day initiation regimen [19]. The inclusion of PP provided an active control with known safety and efficacy; no conclusions about relative efficacy of the 2 treatments can be drawn from the current analysis. Improvements in exploratory efficacy outcomes, including PANSS subscales and CGI-S, were in line with previously reported results for PANSS total score [19]. Decreased caregiver burden, sustained patient satisfaction with medication, and stable quality of life through the outpatient period in AL- or PP-treated patients are consistent with functional improvement that persists over 25 weeks of treatment. Together these exploratory efficacy results indicate that the AL or PP regimens may both improve symptoms of schizophrenia and help address the poor functional outcomes associated with this illness [4, 5].

## Supplementary Information

**Additional file 1.** Supplemental Fig. 1. Patient Flow, ALPINE Study. Supplemental Fig. 2. Medication Satisfaction Questionnaire, Aripiprazole Lauroxil Group. (A) Do you prefer your current injectable or previous oral medication? (B) Rate the level of side effects of current injectable versus previous oral medication. Supplemental Fig. 3. Medication Satisfaction Questionnaire, Paliperidone Palmitate Group. (A) Do you prefer your current injectable or previous oral medication? (B) Rate the level of side effects of current injectable versus previous oral medication.

## Data Availability

The data collected in this study are proprietary to Alkermes, Inc. Alkermes, Inc. is committed to public sharing of data in accordance with applicable regulations and laws, and requests can be submitted to the corresponding author.

## Abbreviations

AL: Aripiprazole lauroxil
AL_NCD_: Aripiprazole lauroxil NanoCrystal® Dispersion
ALPINE: Aripiprazole Lauroxil and Paliperidone palmitate: INitiation Effectiveness
BL: Baseline
CGI-S: Clinical Global Impression−Severity
LAI: Long-acting injectable
MSQ: Medication satisfaction questionnaire
PANSS: Positive and Negative Syndrome scale
PP: Paliperidone palmitate
PRO: Patient-reported outcome
Q-LES-Q-SF: Quality of Life Enjoyment and Satisfaction Questionnaire Short form
