# Supplementary material for: Aripiprazole lauroxil 2-month formulation with 1-day initiation in patients hospitalized for an acute exacerbation of schizophrenia: exploratory efficacy and patient-reported outcomes in the randomized controlled ALPINE study
Source: BMC Psychiatry. 2021 Oct 8;21:492. doi: 10.1186/s12888-021-03420-x (PMC8501701; doi:10.1186/s12888-021-03420-x)
Supplement: Supplementary file 1 — Additional file 1. Supplemental Fig. 1. Patient Flow, ALPINE Study. Supplemental Fig. 2. Medication Satisfaction Questionnaire, Aripiprazole Lauroxil Group. (A) Do you prefer your current injectable or previous oral medication? (B) Rate the level of side effects of current injectable versus previous oral medication. Supplemental Fig. 3. Medication Satisfaction Questionnaire, Paliperidone Palmitate Group. (A) Do you prefer your current injectable or previous oral medication? (B) Rate the level of side effects of current injectable versus previous oral medication. [file 12888_2021_3420_MOESM1_ESM.docx]

**Supplemental Material**

**Aripiprazole Lauroxil 2-Month Formulation With 1-Day Initiation in Patients Hospitalized for an Acute Exacerbation of Schizophrenia: Exploratory Efficacy and Patient-Reported Outcomes in the Randomized Controlled ALPINE Study**

Henry A. Nasrallah, MD; Peter J. Weiden, MD; David P. Walling, PhD; Yangchun Du, PhD; Baiyun Yao, PhD; Sergey Yagoda, MD, PhD; Amy Claxton, PhD

**Contents**

**Supplemental Figure 1.** Patient Flow, ALPINE Study

**Supplemental Figure 2.** Medication Satisfaction Questionnaire, Aripiprazole Lauroxil Group. (**A)** Do you prefer your current injectable or previous oral medication? (**B**) Rate the level of side effects of current injectable versus previous oral medication^.^

**Supplemental Figure 3.** Medication Satisfaction Questionnaire, Paliperidone Palmitate Group. (**A)** Do you prefer your current injectable or previous oral medication? (**B**) Rate the level of side effects of current injectable versus previous oral medication.

**Supplemental Figure 1.** Patient Flow, ALPINE Study


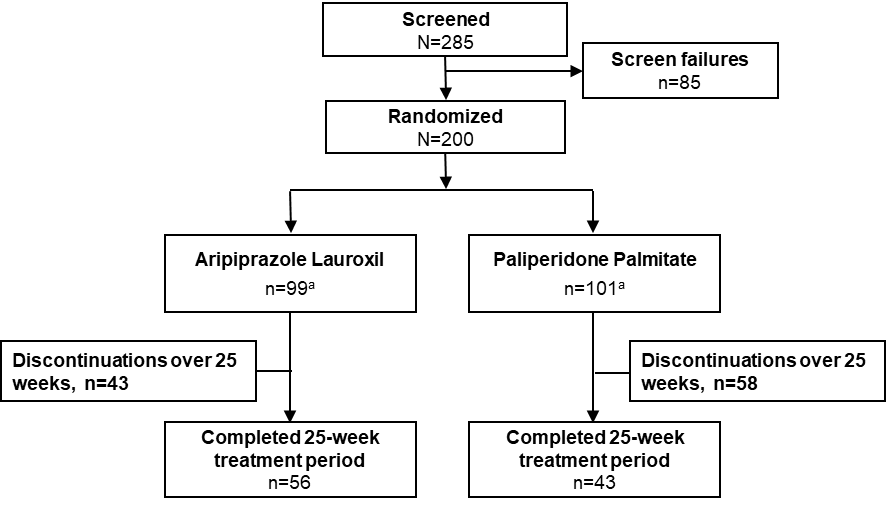


^a^Includes all patients with ≥1 dose of study drug. Five patients (aripiprazole lauroxil, n=3; paliperidone palmitate, n=2) did not receive postbaseline efficacy assessments.

**Supplemental Figure 2.** Medication Satisfaction Questionnaire, Aripiprazole Lauroxil Group

**A.** Do you prefer your current injectable or previous oral medication?^a^

^
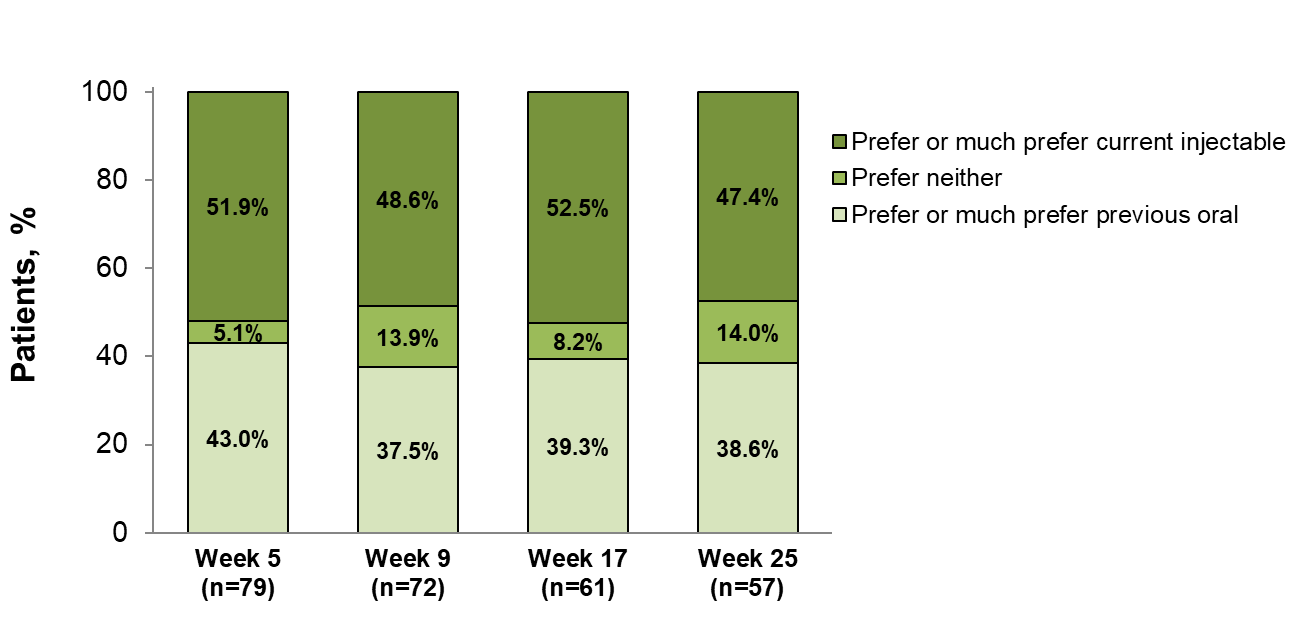
^

**B.** Rate the level of side effects of current injectable versus previous oral medication^b^


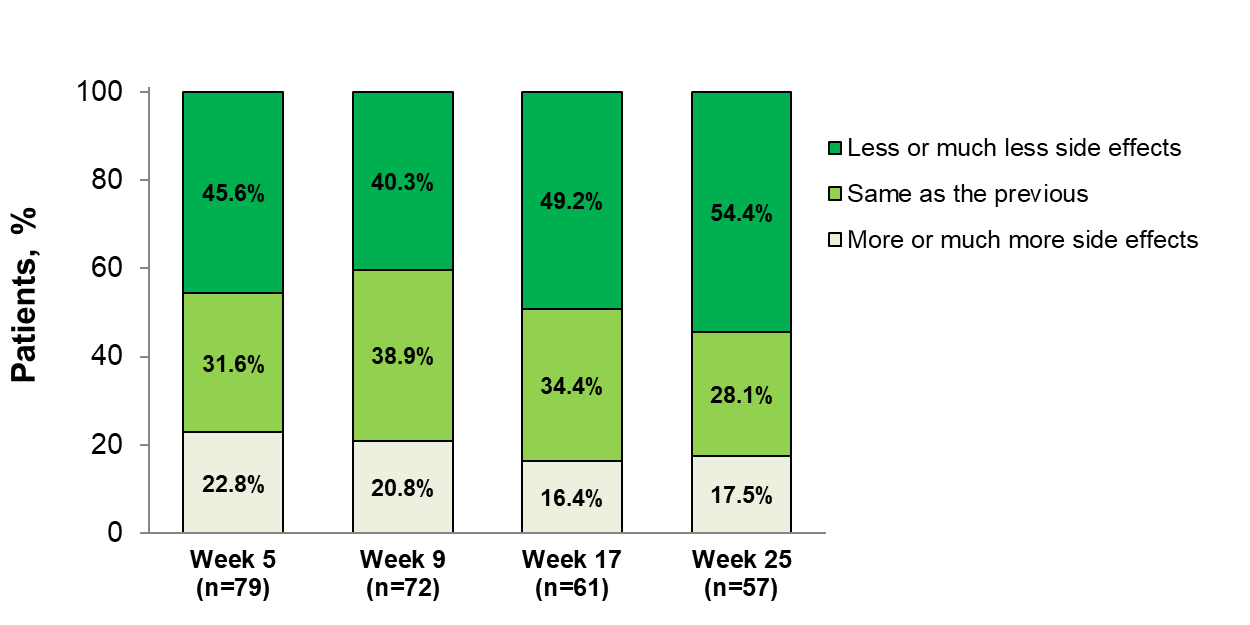


^a^Possible responses: much prefer previous oral, prefer previous oral, neither prefer previous oral nor current injection, prefer current injectable, or much prefer current injectable.

^b^Data labels do not sum to 100% at some timepoints due to rounding.

^c^Possible responses: much less side effects, less side effects, the same as previous, more side effects, much more side effects.

**Supplemental Figure 3.** Medication Satisfaction Questionnaire, Paliperidone Palmitate Group

**A.** Do you prefer your current injectable or previous oral medication?^a^

^
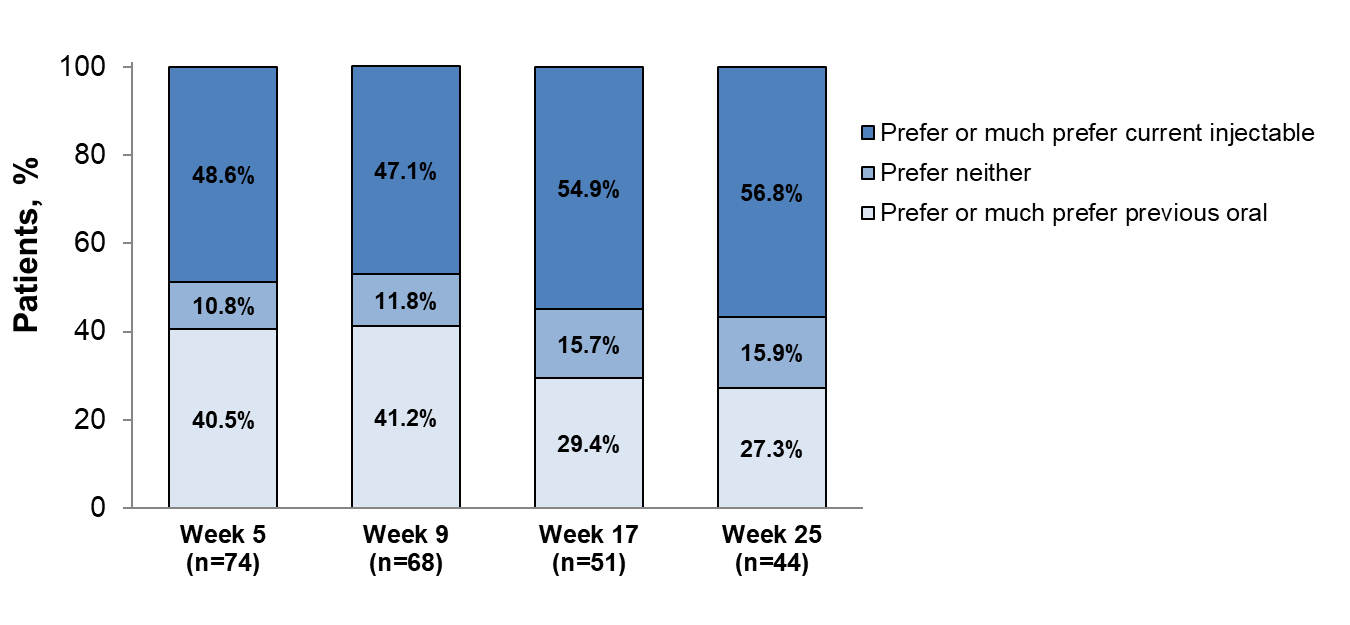
^

**B.** Rate the level of side effects of current injectable versus previous oral medication^b^


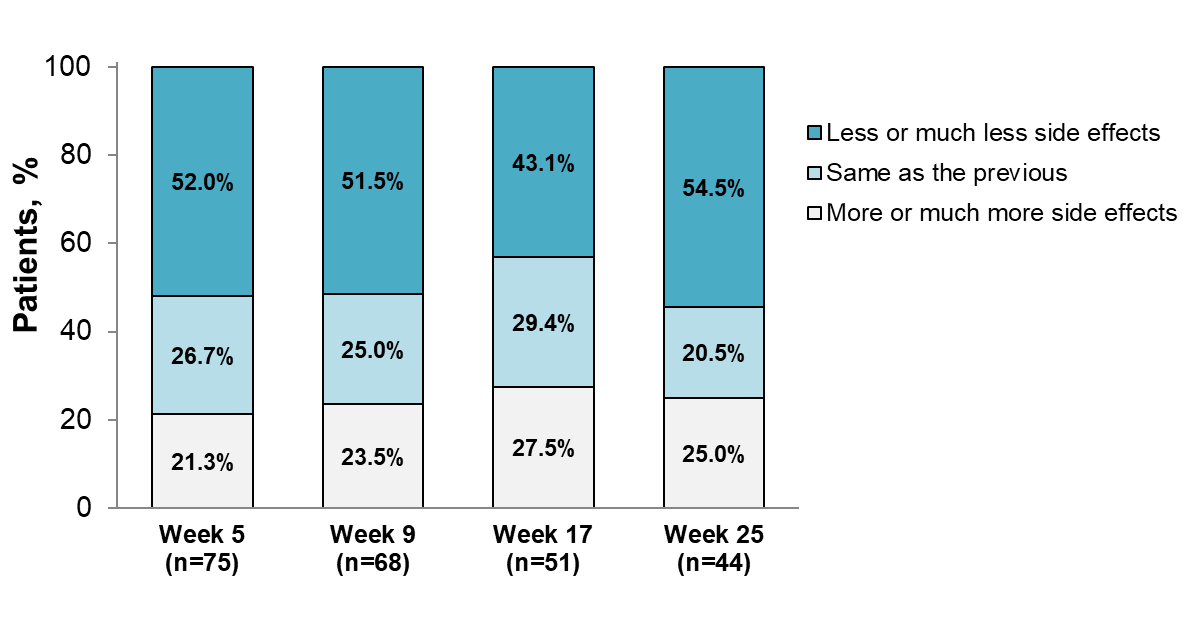


^a^Possible responses were much prefer previous oral, prefer previous oral, neither prefer previous oral nor current injection, prefer current injectable, or much prefer current injectable.

^b^Possible responses were much less side effects, less side effects, the same as previous, more side effects, much more side effects.
